# Domain definition and preliminary functional exploration of the endonuclease NOBP-1 in *Strongyloides stercoralis*

**DOI:** 10.1186/s13071-023-05940-9

**Published:** 2023-11-03

**Authors:** Huan Zhou, Wang Yuan, Weiqiang Lei, Taoxun Zhou, Peixi Qin, Biying Zhang, Min Hu

**Affiliations:** 1 State Key Laboratory of Agricultural Microbiology, College of Veterinary Medicine, Huazhong Agricultural University, Wuhan, China; 2 National Key Laboratory of Cotton Bio-Breeding and Integrated Utilization, School of Life Sciences, Henan University, Kaifeng, China; 3 College of Animal Science and Technology, Jinling Institute of Technology, Nanjing, 210038 China

**Keywords:** *Strongyloides stercoralis*, *Nobp-1*, Parasitic nematode, Transgenesis, Development

## Abstract

**Background:**

Ribosome biogenesis is the process of assembling ribosome complexes that regulate cell proliferation and differentiation with potential regulatory effects on development. Many factors regulate ribosome biological processes. Nin one binding protein (Nob1) has received widespread attention as key genes regulating ribosome biogenesis—the 3ʹ end of the 20S rRNA is cleaved by Nob1 at cleavage site D to form 18S rRNA, generating translationally capable 40S subunit. As a ribosome biogenesis factor, Nob1 may regulate the development of organisms, but almost nothing is known about the function of Nob1 for any parasitic nematode. We explored the functional role of NOBP-1 (the homologous gene of Nob1) encoding gene from a parasitic nematode—*Strongyloides stercoralis*.

**Methods:**

The full-length cDNA, gDNA and promoter region of *Ss-nobp-1* was identified using protein BLAST in WormBase ParaSite according to the *Caenorhabditis elegans* NOBP-1 sequence to analyze the gene structure. RNA sequencing (RNA-seq) data in wormbase were retrieved and analyzed to assess the transcript abundance of *Ss-nobp-1* in seven developmental stages of *S. stercoralis*. The standard method for gonadal microinjection of constructs was carried out to determine the anatomic expression patterns of *Ss-nobp-1*. The interaction between *Ss*-NOBP-1 and partner of NOBP-1 (*Ss-*PNO-1) was assessed by yeast two-hybridization and bimolecular fluorescence complementarity (BiFC) experiments.

**Results:**

The NOBP-1 encoding gene *Ss-nopb-1* from the zoonotic parasite *S. stercoralis* has been isolated and characterized. The genomic DNA representing *Ss-nobp-1* includes a 1599-bp coding region and encodes a protein comprising 403 amino acids (aa), which contains conserved PIN domain and zinc ribbon domain. RNA-seq analysis revealed that *Ss-nobp-1* transcripts are present throughout the seven developmental stages in *S. stercoralis* and have higher transcription levels in iL3, L3 and P Female. *Ss-nobp-1* is expressed mainly in the intestine of transgenic *S. stercoralis* larvae, and there is a direct interaction between *Ss*-NOBP-1 and *Ss*-PNO-1.

**Conclusions:**

Collectively, *Ss*-NOBP-1 has a potential role in embryo formation and the infective process, and findings from this study provide a sound foundation for investigating its function during the development of parasitic nematode.

**Graphical Abstract:**

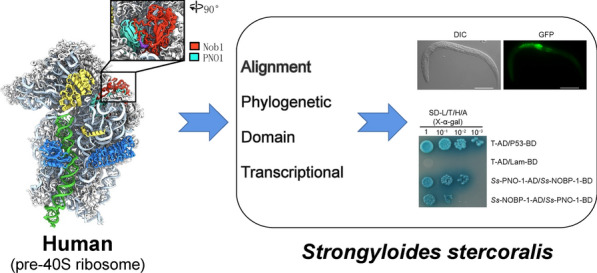

**Supplementary Information:**

The online version contains supplementary material available at 10.1186/s13071-023-05940-9.

## Background

Nematodes are one of the most abundant Metazoa in the animal kingdom. Most parasitic nematodes have a short reproductive cycle and strong reproductive ability and cause serious harm to the host [1–3] and major economic losses to the breeding industry, greatly restricting the healthy development of animal husbandry [4–7]. *Strongyloides stercoralis* is a parasitic nematode of humans and dogs, affecting the health of an estimated 600 million people worldwide [8]. The life history of *S. stercoralis* includes in vivo parasitic stage and in vitro free-living stage [3]. Infective third-stage larvae (iL3s) invade somatic tissues and migrate to the host intestine to develop into parasitic female adults (P Female). P Females lay sexually differentiated eggs by mitotic parthenogenesis. These eggs hatch in the intestine, and the post-parasitic first-stage larvae (PP L1s) are either excreted through feces into the free life stage, or develop to auto-infective L3s (aL3s) directly in the host intestine and continue to produce a new generation of parasitic female adults (P female) [3]. The free-living stage contains the complete process of larval to adult development, the female and male adult worms can mate, and the eggs laid can hatch and develop into infectious tertiary larvae in vitro [3]. This species of parasitic nematode is morphologically similar to the free-living nematode *Caenorhabditis elegans*, and transgenic techniques have been successfully applied in this parasite [9]. Molecular biology studies of *S. stercoralis* will compensate for the limitations of *C. elegans* as a model organism in parasitic nematode research.

Ribosome biogenesis is the process of assembling ribosome complexes [10], a highly coordinated process closely related to protein synthesis, cell proliferation, differentiation and apoptosis [11] and also regulating development by influencing the translation of mRNA [12]. The early stages of assembly and maturation of the ribosomal small subunit (SSU) are mediated by the SSU processome [13]. In the final steps of cytoplasmic maturation of the pre-40S, the 3ʹ end of the 20S rRNA is cleaved by nuclease Nob1 at cleavage site D to form 18S rRNA and further generating translationally capable 40S subunit [14–17]. Nob1 contains the PilT N-terminal (PIN) domain [14, 16], which is common to exonuclease or endonuclease [18, 19] and zinc ribbon domain [20–22]. PNO-1 (partner of Nob1p) is the interaction partner of the endonuclease Nob1; it regulates the enzymatic function of Nob1 and together with Nob1 mediates the cleavage of 20S rRNA into mature 18S rRNA [23]. The structure of Nob1 binding to 40S pre-rRNA has not been resolved, and it is speculated that the binding of Nob1 to rRNA is flexible, and the interaction between PNO-1 and the rRNA of the platform is close to the 40S “neck” [24]. In the process of ribosome biogenesis, the functions of Nob1 and PNO-1 are complementary [23].

Ribosome biogenesis regulates cell proliferation and differentiation [11] and has a potential regulatory effect on the development of organisms [12]. Nob1 has received widespread attention as key genes regulating ribosome biogenesis [14, 15, 17], and it is speculated that Nob1 may regulate the development of organisms according to the functional studies of ribosome biogenesis, but just a few studies have been done on the regulation of development, and almost nothing is known about the function of Nob1 for any parasitic nematode. In the present study, we explored NOBP-1 (the homologous gene of NOB-1) encoding gene *Ss-nobp-1* of *S. stercoralis* and investigated its temporal and spatial expression patterns towards the ultimate goals of uncovering its function and understanding its role in *S. stercoralis*, which facilitate the discovery of new interventions to control nematodiases.

## Methods

### Ethics statement

According to the protocol (permit no. SYXK-2015-0029) approved by the Animal Ethics and Animal Experimentation Committee of Hubei Province, the *S. stercoralis* (UPD strains) were maintained in steroid-treated beagle dogs. The dogs were fed standard laboratory chow which complied with guidelines proposed by the Administration of Affairs Concerning Experimental Animals of PR China.

### Parasite culture

*Strongyloides stercoralis* was propagated in beagles treated with prednisolone acetate [25]. Free-living adult parasites were collected from 48-h-old coprocultures (22 °C) via the Baermann funnel technique. Afterward, adult worms were washed with sterile buffered saline (BU) and then cultured with *Escherichia coli* OP50 on Nematode Growth Medium (NGM) plates before being injected with indicated constructs.

### Genomic DNA and RNA extraction

Genomic DNA was extracted from 10,000–20,000 iL3s (infective third-stage larvae) using an EasyPure Genomic DNA kit (TransGen Biotech, Beijing, China) and used immediately or stored at − 20 °C until use. Total RNA was extracted from ~ 25,000 iL3s by TRizol reagent extraction according to the manufacturer’s instructions (Life Technologies, USA). A complementary DNA (cDNA) was amplified using PrimeScript First-Strand cDNA Synthesis Kit (Takara, Beijing, China) and stored at − 80 °C until further analysis.

### Bioinformatic and phylogenetic analyses

The sequence of *Ce-nobp-1* (WS286, code Y75B8A.2) was obtained from Wormbase (https://wormbase.org/), and the homologues of Nob1 in *S. stercoralis* were identified using protein BLAST in WormBase ParaSite (https://parasite.wormbase.org/Tools/Blast) according to the *C. elegans* protein sequence. The amino acid sequences of *Ss*-NOBP-1 were downloaded directly.

The amino acid sequences inferred from *Ss*-NOBP-1 and 11 selected sequences from homologues in other species were subjected to phylogenetic analysis by MEGA7 and manually adjusted. The 11 homologous sequences of *Ss*-NOBP-1 were retrieved from four nematodes [*C. elegans* (NP_491090.1), *Ascaris suum* (GS_18771), *Loa loa* (XP_020302808.1), *Brugia malayi* (XP_001899836.1)] and seven non-nematodes [*Homo sapiens* (NP_054781.1), *Pan troglodytes* (XP_523405.1), *Canis lupus familiaris* (XP_546853.2), *Xenopus tropicalis* (NP_001016830.1), *Danio rerio* (XP_683754.2), *Drosophila melanogaster* (NP_572603.1), *Saccharomyces cerevisiae S288C* (NP_014699.1)]. The sequence of *Ss*-NOBP-1 was aligned with Nob1 amino acid sequences of *H. sapiens* and other seven species using BioEdit to identify and designate functional domains respectively; these domains were labeled using Photoshop CS 6.0.

### Transcriptional analysis

The raw data of RNA-seq from isolated larvae at different stages were retrieved from http://www.ebi.ac.uk/arrayexpress/ [26, 27]. The seven developmental stages of the PV001 line (derived from a single female worm of the UPD line) of *S. stercoralis* were used for transcriptional profiling of *Ss-nobp-1*, including free-living females (FL Female), post-free-living first-stage larvae (PFL L1), iL3, in vivo activated third-stage larvae (L3+), parasitic females (P Female), post-parasitic first-stage larvae (PP L1) and post-parasitic third-stage larvae (PP L3). The specific transcript abundance of *Ss-nobp-1* was calculated as fragments per kilobase of coding exon per million fragments mapped (FPKM), and the FPKM values of the coding sequence (CDS), ± 95.0% confidence interval, were calculated using Cuffdiff v.2.0.2 (http://cufflinks.cbcb.umd.edu/).

### Transformation constructs and transformation of *S. stercoralis*

The *Ss-nobp-1* promoter sequence amplified from gDNA of *S. stercoralis* with *Ss-nobp-1*-Prom-F/R primers (Additional file 1: Table S1) was subcloned upstream of *gfp* between *Sma*I and *Age*I restriction sites in the promoter-less vector pAJ01 plasmid [28] to create the plasmid pAJ01*-Ss-nobp-1*. The construct was diluted to 50 ng/μl and stored at − 20 °C for microinjection.

The standard method for gonadal microinjection of constructs was carried out as described previously [9, 25]. The transformed female worms were transferred onto a new NGM plate seeded with *E. coli* OP50, and each female was mated with two males on the same plate. The transgenic F1 larvae expressing GFP were screened using a stereomicroscope (SZX16 Olympus). Subcellular and tissue-specific distribution of *Ss*-NOBP-1 isoform was examined by Olympus BX53 microscope [29, 30]. In brief, worms were transferred to a 2.0% agarose pad (Lonza) containing 100 mM levamisole solution (Sigma, Aldrich) for immobilization and then placed onto a microslide for imaging by an Axiocam 503 mono-camera.

### Yeast two-hybrid assay

For yeast two-hybrid assays, the coding regions of *Ss*-NOBP-1 (403 amino acid residues, from 1–403aa) and *Ss*-PNO-1 (260 amino acid residues, from 1–260aa) were amplified from cDNA by PCR with corresponding primers listed in Additional file 1: Table S1. The amplified fragments were subcloned into the plasmid pGBKT7 (Clontech), which contains the GAL4 DNA-binding domain, producing the constructs pGBKT7-*Ss*-NOBP-1 and pGBKT7-*Ss*-PNO-1. The coding regions of *Ss*-NOBP-1 (1–403aa) and *Ss*-PNO-1 (1–260aa) were PCR amplified from cDNA using corresponding primers listed in Additional file 1: Table S1 and cloned into the pGADT7 vector, which contains the GAL4 activation domain, creating the plasmids pGADT7-*Ss*-NOBP-1 and pGADT7-*Ss*-PNO-1.

To test autoactivation of *Ss*-NOBP-1 and *Ss*-PNO-1 proteins, all four plasmids were transformed into the yeast strain Y190 (Clontech) with the empty pGBKT7 or pGBKT7 plasmid, respectively. Transformants were then grown on plates containing the minimal yeast medium without tryptophan (SD-L/T), or SD-L/T/H supplemented with X-α-Gal (SD/-Trp/X), or SD-L/T/H supplemented with X-α-Gal and aureobasidin A (SD-L/T/H/A). Lack of autoactivation was indicated by white colonies on SD-L/T and SD-L/T/H plates and absence of colony growth on SD-L/T/H/A plates. Yeast two-hybrid interaction assays were performed according to the manufacturer’s instructions (Clontech, USA). Competent cells of *S. cerevisiae* strain Y190 (Clontech) were transformed simultaneously with pGBKT7-*Ss*-NOBP-1 or pGBKT7-*Ss*-PNO-1 and pGADT7-*Ss*-NOBP-1 or pGADT7-*Ss*-PNO-1. Yeast cells that had been cotransformed with pGBKT7-Lam (for human lamin C protein) and pGADT7-T were used as negative control, whereas those cotransformed with pGBKT7-P53 (for murine p53 protein) and pGADT7-T were used as positive control.

### Bimolecular fluorescence complementation (BiFC)

The sequence of *Ss*-NOBP-1 (1–430aa), which was amplified with the primers *Ss*-NOBP-1-HA-F/R (Additional file 1: Table S1), was inserted in pbJun-HA-KN151 between *Nhe*I and *Xho*I restriction sites to generate p*Ss*-NOBP-1-HA-KN151. Equally, the sequence of *Ss*-PNO-1 (1–260aa), which was amplified with the primers *Ss*-PNO-1-Myc-F/R (Additional file 1: Table S1), was inserted in pbFos-Myc-LC151 between *Nhe*I and *Pvu*I restriction sites to generate p*Ss*-PNO-1-Myc-LC151. Plasmids were extracted from *E. coli* and stored at − 20 °C.

To examine the interaction between *Ss*-NOBP-1 and *Ss*-PNO-1, p*Ss*-NOBP-1-HA-KN151 and p*Ss*-PNO-1-Myc-LC151 were co-transfected at a ratio of 1:1 (0.25 μg each) into NIH/3T3 cells, which were seeded onto 24-well plates and grown to about 70.0% confluency using FuGENE® HD Transfection Reagent (Promega). Cells were incubated at 37 °C for 48 h and subsequently imaged using the fluorescent microscope at 543 nm (Nikon ECLIPSE Ti2, Japan). Constructs expressing bJun (pbJun-HA-KN151) and bFos (pbFos-Myc-LC151) were co-transfected as a positive control; pMyc-LC151 and pHA-KN151 were co-transfected and served as the negative control.

## Results

### Genomic organization of *S. stercoralis* nobp-1

**Fig. 1:**
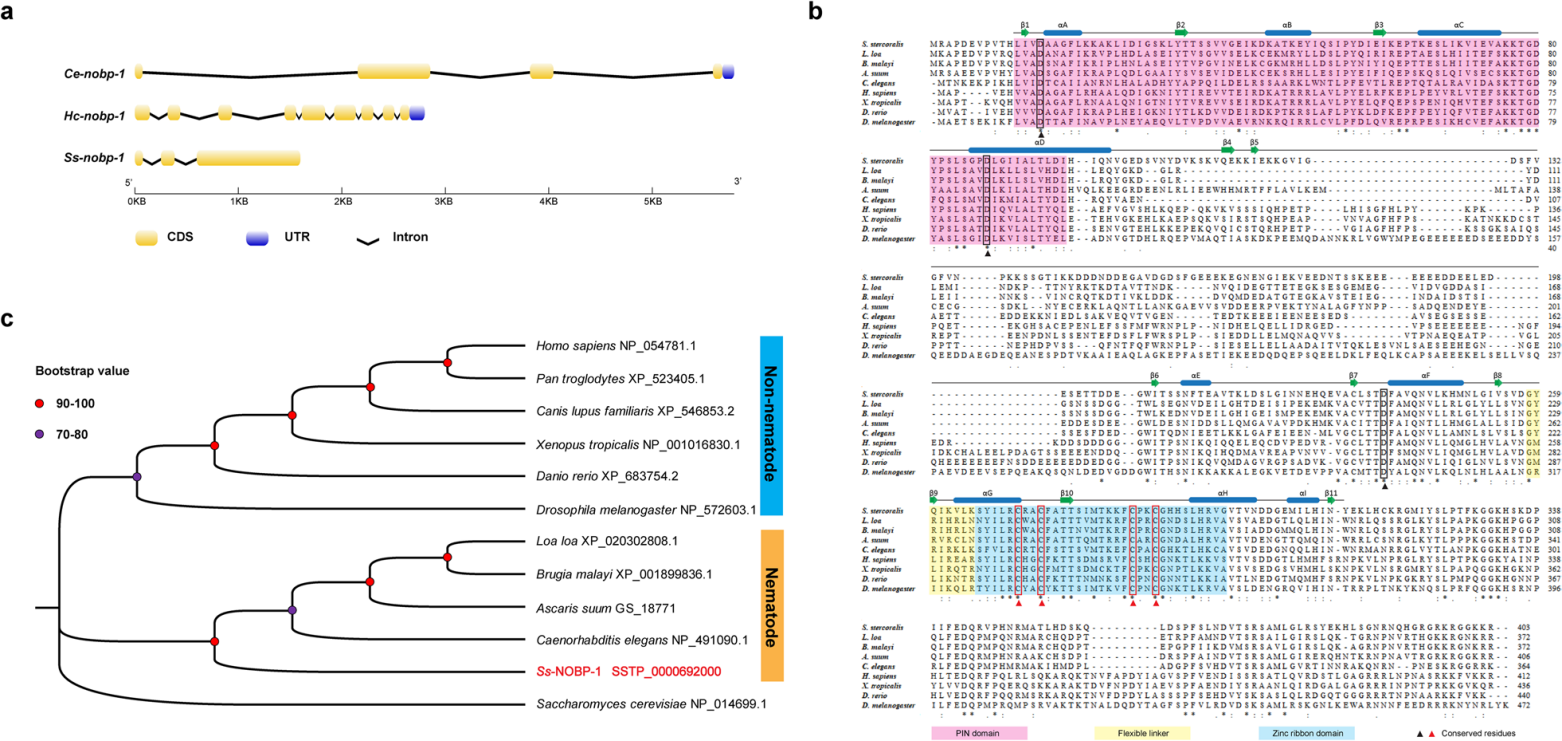
*Ss*-NOBP-1 displays conserved features with homologs from selected species. **a** The structures of the *nobp-1* genes of *Strongyloides stercoralis*, *Caenorhabditis elegans* and *Haemonchus contortus.* **b** Alignment of the amino acid sequences of *S. stercoralis* NOBP-1 with homologs from eight other species. The eight selected species are *Loa loa* (XP_020302808.1), *Brugia malayi* (XP_001899836.1), *Ascaris suum* (GS_18771), *C. elegans* (NP_491090.1), *Homo sapiens* (NP_054781.1), *Xenopus tropicalis* (NP_001016830.1), *Danio rerio* (XP_683754.2), *Drosophila melanogaster* (NP_572603.1). Functional domains including PIN domain (pink), flexible linker (yellow) and zinc ribbon domain (blue) are highlighted, and the conserved aspartate residues (black) and cysteine residues (red) are marked with solid wireframes. The blue box and green arrows above the sequence represent the α-helices and β-strands. **c** The phylogenetic tree of *S. stercoralis* NOBP-1 with homologues from 10 selected species. These species contain four nematode species, one insect, two fish and amphibian species and three mammalian species. The Nob1 from *Saccharomyces cerevisiae* (NP_014699.1) is used as the outgroup. Accession numbers for the sequences listed follow the species name. Bootstrap values are displayed in the tree

The structures of the *nobp-1* gene of *S. stercoralis*, *C. elegans* and *Haemonchus contortus* were obtained by aligning their genomic DNA sequences with respective cDNA sequences. Comparison of cDNA and genomic DNA sequences of *Ss-nobp-1* revealed that there are two introns in the coding sequence (Fig. 1a). The genomic DNA representing *Ss-nobp-1* (WormBase ParaSite Accession No. SSTP_0000692000) is 1599 bp long and encoded a protein comprising 403aa. Comparison of the *Ss-nobp-1* gene structure with its homologs of *C. elegans* and *H. contortus* showed that *Ss-nobp-1* contained the fewest exons and the shortest total length of introns: three exons of 78–998 bp and two introns of 174–213 bp (Fig. 1a).

### Structural features and sequence analysis of *Ss*-NOBp-1

The amino acid sequence of *Ss*-NOBP-1 was aligned to homologs from eight selected species, including four nematodes and four non-nematode species (Fig. 1b). The alignment showed that *Ss*-NOBP-1 contained a conserved PIN domain and a zinc ribbon domain as well as a flexible adapter between the two domains (Fig. 1b). The PIN domain contains two aspartate residues (D15 and D88) that conserve regulation of Nob1 ribosome function [14] and an aspartate residue (D239) outside the PIN domain that is conserved in archaea and involved in the binding of Mn2+ to Nob1 [31]. In addition, four cysteine residues that regulate the metal ion binding in the highly conserved zinc ribbon domain are conserved in *Ss*-NOBP-1 (C271, C274, C286 and C289) (Fig. 1b) [32].

Phylogenetic analyses of the full-length amino acid sequences of *Ss*-NOBP-1 and 11 other Nob1 homologs representing a range of different species showed that there is a concordance in topology among the MP, ML and NJ trees. *Ss*-NOBP-1 clustered with four Nob1s from other nematode species with a nodal support of 94.0%; all nematode Nob1s formed a cluster with absolute support to the exclusion of seven Nob1s from non-nematode species (Fig. 1c).

### Transcriptional analysis of *Ss*-nobp-1

**Fig. 2:**
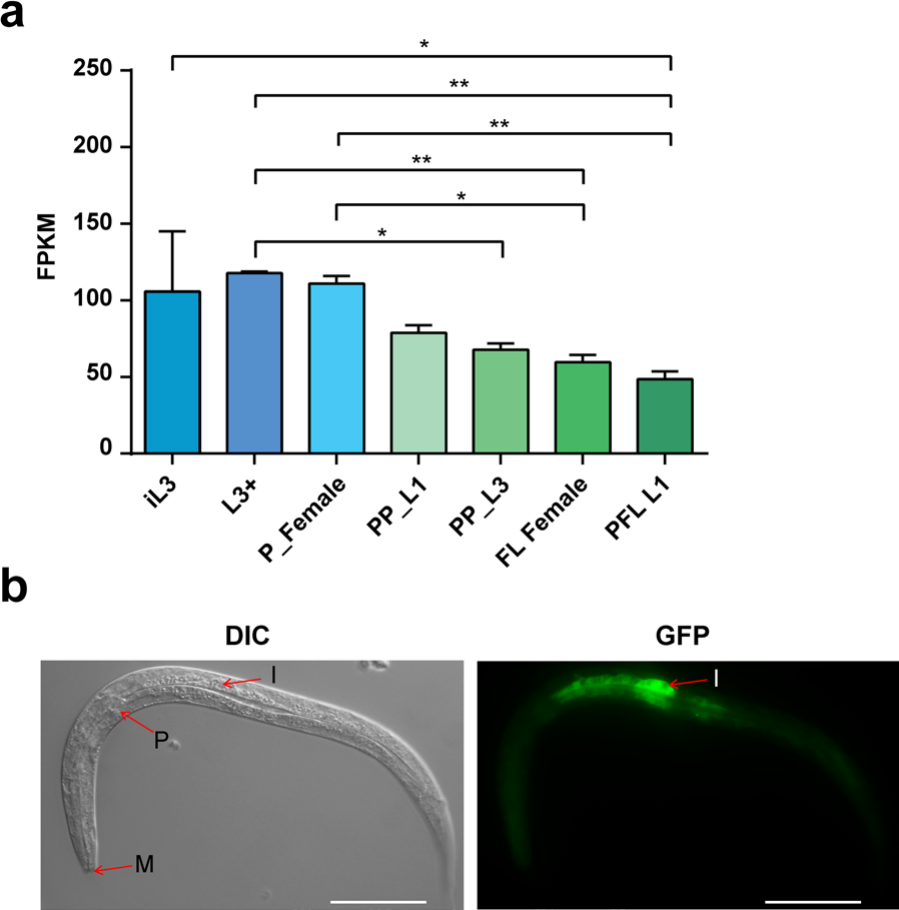
Anatomic expression pattern of *Ss*-NOBP-1 in *Strongyloides stercoralis*.** a** Transcriptional profiles of *Ss-nobp-1* in *S. stercoralis*. Seven developmental stages examined were: infectious third-stage larvae (iL3), in vivo activated third-stage larvae (L3+), parasitic females (P Female), post-parasitic first-stage larvae (PP L1), post-parasitic third-stage larvae (PP L3), free-living females (FL Female) and post free-living first-stage larvae (PFL L1). Transcript abundances are expressed as fragments per kilobase of coding exon per million mapped reads (FPKM). * and ** indicate *P* < 0.01 and *P* < 0.001. **b** Differential interference contrast (DIC) and fluorescence (GFP) images showing the expression of GFP under the putative *Ss-nobp-1* promoter in transgenic *S. stercoralis* larvae in the intestine. I, intestine; P, pharynx; M, mouth. Scale bar = 50 μm

RNAseq revealed that *Ss-nobp-1* is transcribed in all developmental stages of *S. stercoralis* examined (Fig. 2a). *Ss-nobp-1* has relatively uniform transcript levels at multiple developmental stages, and the abundance of these transcripts decreases during the transition from iL3 to PFL L1. After iL3 infects the host, it develops and reproduces with the ordinate in Fig. 2a; the *Ss*-*nobp-1* transcript remains high during the process of iL3 entering the body and developing into P Female. During the development of PP-L1, which is produced by P Female, to FL Female, the *Ss*-*nobp-1* transcript decreases continuously and is lowest in the offspring larvae PFL L1 (Fig. 2a). The transcript abundance of *Ss*-*nobp-1* decreases with larval growth and development in the ordinate of Fig. 2a, and its transcript increases sharply to a higher level during the development of PFL L1 to infectious iL3, ultimately remaining at a high level in the activated L3+ (Fig. 2a).

### Anatomic expression pattern of *Ss*-NOBP-1

To investigate the anatomical expression pattern of *Ss*-NOBP-1 in vivo, we transformed parental free-living *S. stercoralis* females with constructs pAJ01*-Ss-nobp-1* (*gfp* expression under control of the 1312 bp putative *Ss-nobp-1* promoter), and transformed females were paired with double numbers of wild-type *S. stercoralis* males. In the free-living females injected with pAJ01*-Ss-nobp-1*, some immature eggs have GFP expression in the uterus of free-living females (data not shown); 48 h after transformation, the PFL L1 progeny were screened for GFP fluorescence. A total of 10 GFP-expressing larvae from two injection experiments were analyzed. GFP expression under the *Ss-nobp-1* promoter predominated in the intestine of transgenic PFL L1s and PFL L2s (Fig. 2b), and a stronger fluorescence signal in the anterior part of the intestine appeared in 60% of GFP-expressing larvae. In addition, GFP expression in the subcutaneous tissue was found in several transgenic larvae at 72 h after micro-injection. No fluorescence was observed in non-transgenic worms.

### *Ss*-NOBp-1 interact with *Ss*-PNO-1

**Fig. 3:**
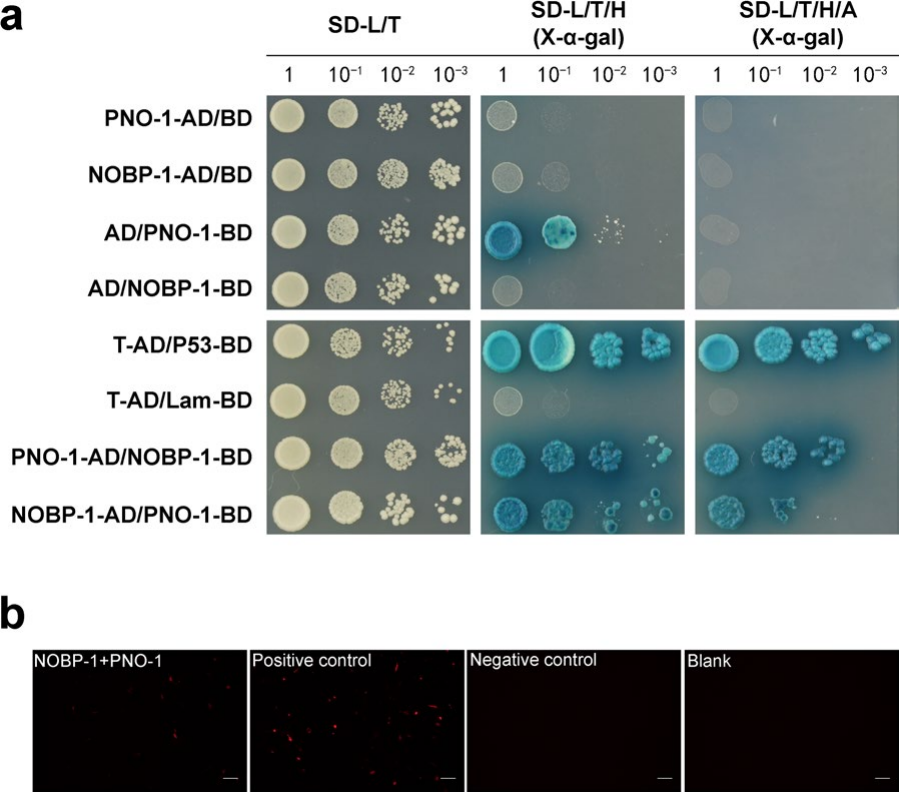
*Ss*-NOBP-1 interacts with *Ss*-PNO-1.** a** *Ss*-NOBP-1 interacts with *Ss*-PNO-1 in the yeast two-hybrid system. Yeast (*Saccharomyces cerevisiae* strain Y190) containing pGADT7-*Ss*-NOBP-1 as bait and pGBKT7-*Ss*-PNO-1 as prey, or pGADT7-*Ss*-PNO-1 as bait and pGBKT7-*Ss*-NOBP-1 as prey, was grown for 48 h on synthetic defined (SD) medium that lacked amino acid (Trp, Leu, His or Ade) and was assayed for LacZ expression for α-galactosidase activity (α-gal). The vectors pGBKT7-Lam and pGADT7-T were used as negative control. pGBKT7-P53 and pGADT7-T were used as positive control. Blue color indicates interaction. **b** BiFC experiment was performed to check the interaction between *Ss*-PNO-1 and *Ss*-NOBP-1 in mammalian cells. *Ss*-NOBP-1 fused to KN151 and *Ss*-PNO-1 fused to LC151 were coexpressed in NIH/3T3 cells. pbJun-HA-KN151 and pbFos-Myc-LC151 were coexpressed as a positive control. pHA-KN151 and pMyc-LC151 as a negative control and Blank have no transfection control cells. Punctate red fluorescence (BiFC signal) in the cell indicates interaction. Scale bar = 100 μm

PNO-1 is the interaction partner of the endonuclease Nob1, and the interaction of PNO-1 and Nob1 mediates the cleavage of 20S rRNA into mature 18S rRNA. To check the interaction between *Ss*-NOBP-1 and *Ss*-PNO-1, the point-to-point yeast two-hybrid assay was performed. Yeast transfected with *Ss*-NOBP-1 or *Ss*-PNO-1 constructs could be grown normally on the medium without visible effects on its growth. In addition, the four transfection constructs (pGADT7-*Ss*-NOBP-1, pGADT7-*Ss*-PNO-1, pGBKT7-*Ss*-NOBP-1 and pGBKT7-*Ss*-PNO-1) were co-transfected with no inserts pGBKT7 or pGBKT7 plasmid, respectively, and the yeast strains could be grown on SD-L/T/H medium. None of the other combinations produced blue signal except for pGBKT7-*Ss*-PNO-1. Also, none of the combined yeasts grew or produced a blue signal on SD-L/T/H/A medium (Fig. 3a). Co-transfection of pGADT7-*Ss*-NOBP-1 and pGBKT7-*Ss*-PNO-1 or pGADT7-*Ss*-PNO-1 and pGBKT7-*Ss*-NOBP-1 produces a distinct blue signal on SD-L/T/H and SD-L/T/H/A media, while both negative (pGBKT7-Lam and pGADT7-T) and positive (pGBKT7-P53 and pGADT7-T) controls work properly (Fig. 3a).

BiFC experiment was performed for direct observation of the interaction between *Ss*-PNO-1 and *Ss*-NOBP-1 in mammalian cells. *Ss*-NOBP-1 fused to KN151 and *Ss*-PNO-1 fused to LC151 were coexpressed in NIH/3T3 cells. After incubation at 37 °C for 48 h, cells cotransfected with p*Ss*-NOBP-1-HA-KN151 and p*Ss*-PNO-1-HA-KN151 showed punctate red fluorescence (BiFC signal) in the cell (Fig. 3b). pbJun-HA-KN151 and pbFos-Myc-LC151 were coexpressed as a positive control, and the cells showed bright red fluorescence in the nucleus (Fig. 3b). No BiFC signal was observed in negative control cells or no transfection control cells (Fig. 3b).

## Discussion

Nob1 regulates the cleavage of D site during ribosome small subunit maturation. In addition, it is also involved in proteasome function, although no domains are known to be associated with proteasome function [33]. In addition, Nob1 is a potential biomarker in cancer, and the expression of Nob1 in cancer tissues is significantly increased and affects the prognosis of cancer [34]. In the present study, we isolated and characterized the Nob1 coding gene *Ss-nobp-1* of *S. stercoralis*, an important parasite infecting humans and dogs, and verified the interaction between *Ss*-NOBP-1 and a partner protein of Nob1p; all these laid the foundation for the functional studies of Nob1 in parasitic nematodes.

*Ss*-NOBP-1 contains the PIN domain (PilT N-terminus, from which the name PIN originates) commonly found in exonuclease or endonuclease [35–38]. The nuclease activity of PIN domains was originally proposed by bioinformatic analysis, characterizing the similarity of PIN domain to nucleases of the FLAP protein family [38]. The crystal structure of the archaeal PIN domain shows that the PIN domain has structural similarities to the RNaseH superfamily [18, 37, 39–42], and the acidic residues at the active site of RNaseH are absolutely conserved in PIN domain [43]. These acidic residues coordinate divalent metal ions or have metal-dependent exonuclease or endonuclease activity [18, 19, 32, 37, 44]. In yeast, D15 and D92 residues conserved in the PIN domain are required for Nob1 protein function, and the point mutation in D15 or D92 causes cleavage at the D site to be blocked, which in turn leads to modest accumulation of 20S prerRNA and intense depletion of 18S rRNA [14, 32]. The PIN domain of Nob1 in yeast and the D92 residue therein are conserved in *Ss*-NOBP-1, and it is speculated that the PIN domain of *Ss*-NOBP-1 has similar activity and functions.

Except for the PIN domain, *Ss*-NOBP-1 contains another key functional domain shared by exonuclease or endonuclease—the zinc ribbon domain [45]—which is an anchor point of the protein on the nascent subunit and is located near the cleavage site [32]. While the sequences of Nob1 in archaea and eukaryotes vary widely in length, all identified Nob1 homologs, including *Ss*-NOBP-1, apparently contain a zinc ribbon domain with four highly conserved cysteine residues, which have been shown to regulate the Zn2-ion-binding capacity of the zinc ribbon domain [32]. It is speculated from the conservatism of the functional domains of *Ss*-NOBP-1 and its homologs that *Ss*-NOBP-1 may be involved in the ribosome process of *S. stercoralis* in the same way as its homologs function in yeast and human cells [14–16, 46].

The transcription level of genes changes with the development of *S. stercoralis* [47–49]. *Ss-nobp-1* shows transcriptional differences at different stages of parasite development, and the transcription level is directly related to their functions. *Strongyloides stercoralis* is a parasitic nematode whose life cycle includes free-living and parasitic stages [3]. The abundance of *Ss-nobp-1* transcripts is higher in the parasitic stages (L3+ and P Female) and gradually decreases with the development process in vitro of the host and with the lowest transcription at PFL L1. During this process, the function of *Ss-nobp-1* is likely to gradually decrease as its transcription levels change, which requires more functional studies for later verification. Interestingly, when PFL L1s develop into iL3 larvae, the transcription level of *Ss-nobp-1* increases significantly and further increases as iL3 transforms into L3+ and migrates to the gut to develop into FL after infecting the host. The difference in transcription levels of *Ss-nobp-1* between free-living and parasitic stages indicates that *Ss-nobp-1* has a more important function in the infection process of *S. stercoralis* and in parasitic development stage. Nob1 is essential for the maturation of the ribosome subunit, but the transcriptional level changes of *Ss*-*nobp-1* are not consistent with a variety of ribosomal proteins during parasite development (data not shown), indicating that *Ss-nobp-1* has the same functions as their homologous genes other than ribosome assembly [50].

*Ss*-NOBP-1 is mainly localized in the intestinal tissue of *S. stercoralis*. The intestine of *C. elegans*, which is very similar in morphology to *S. stercoralis* [3], is a simple tube consisting of 20 cells [51]. In addition to its regular digestion and nutrient absorption functions, the intestine also performs the function of the liver, while also playing an important role in pathogen infection, immunity and longevity [52–56]. The lack of intestinal tissue can prevent larvae from surviving [57], because they are unable to either absorb nutrients or defend against the invasion of foreign pathogens. In *S. stercoralis*, functional abnormalities of genes specifically expressed in the gut also affect larval survival [58]. In hermaphrodites of *C. elegans*, the yolk lipoprotein secreted and absorbed by developing oocytes is produced by the intestine [59, 60], and genes specifically expressed in the intestine are important for embryonic development [61]. *Ce*-NOBP-1 is critical for embryonic survival (https://wormbase.org/species/c_elegans/gene/WBGene00003779), and RNAi of *Ce-pno-1* can cause embryonic lethality, maternal sterility, patchy coloration and protruding vulva (https://wormbase.org/species/c_elegans/gene/WBGene00013144). The conservation of Nob1 in *S. stercoralis* and *C. elegans* suggests that *Ss*-NOBP-1 is likely involved in embryo and larval maintenance. In addition, *Ss*-NOBP-1 is similar to *Ss*-RIOK-2, which is also a ribosome assembly factor [24], mainly localized in the intestinal tissue of *S. stercoralis* larvae [47, 61]. The similar expression patterns of *Ss*-NOBP-1 and *Ss*-RIOK-2 are consistent with their functional correlation during ribosome maturation, and it is speculated that *Ss*-NOBP-1s retain the conserved cleavage function at D site of the small ribosomal subunit.

Nob's cleavage of the D site of the pre-rRNA sequence is regulated by PNO-1; Nob1/PNO-1 is the key complex responsible for the final maturation step of the 18S pre-rRNA [23]. PNO-1, a core component of the SSU RRP complex, is required for pre-rRNA processing and binds to pre-rRNAs in the nucleoli and cytoplasm [23, 24]. In humans and yeast, Nob1 and PNO-1 interact with each other directly, and this interaction does not depend on the conserved PIN domain, zinc ribbon domain and non-conserved region at the C-terminus [22]. A short sequence motif that regulates the interaction between Nob1 and PNO-1 in humans has low retention in *S. stercoralis*, but the interaction between the two still exists, indicating that the functions of Nob1 and PNO-1 in *S. stercoralis* are still related and *Ss*-NOBP-1 may play a similar role in ribosome maturation and processing in *S. stercoralis*.

## Conclusion

We have isolated and characterized the *Ss*-NOBP-1 encoding gene *Ss-nobp-1* from the zoonotic parasite *S. stercoralis*. *Ss*-NOBP-1 contains PIN domain and zinc ribbon domain. *Ss-nobp-1* transcript is present throughout development in *S. stercoralis* and has higher transcription levels in iL3, L3 and P Female. Native protein *Ss-*NOBP-1 is expressed mainly in the gut of larvae, and there is a direct interaction between *Ss*-NOBP-1 and *Ss*-PNO-1. Collectively, these findings suggest that *Ss*-NOBP-1 plays an important role in embryo formation and the infective process.

### Supplementary Information

**Additional file 1: Table S1.** Oligonucleotide primers used in the present study.

## Data Availability

The data supporting the conclusions of this article are provided within the article.
