# Supplementary material for: Domain definition and preliminary functional exploration of the endonuclease NOBP-1 in Strongyloides stercoralis
Source: Parasit Vectors. 2023 Nov 3;16:399. doi: 10.1186/s13071-023-05940-9 (PMC10623843; doi:10.1186/s13071-023-05940-9)
Supplement: Supplementary file 1 — Additional file 1: Table S1. Oligonucleotide primers used in the present study. [file 13071_2023_5940_MOESM1_ESM.docx]

**Additional information**

**Additional file 1: Table S1.** Oligonucleotide primers used in the present study.

| Name | Sequence (5’-3’) |
| --- | --- |
| pGBKT7-*Ss*-NOBP-1-F | TCAGAGGAGGACCTGCATATGATGAGAGCTCCAGATGAGGTGC |
| pGBKT7-*Ss*-NOBP-1-R | CCGCTGCAGGTCGACGGATCCTTATCTCTTCTTACCACCACGTTTTC |
| pGBKT7-*Ss*-PNO-1-F | TCAGAGGAGGACCTGCATATGATGGCAGATATGGATATACCAATGG |
| pGBKT7-*Ss*-PNO-1-R | CCGCTGCAGGTCGACGGATCCTTATTTGTCTTGTATCGTATTATTAC |
| pGADT7-*Ss*-NOBP-1-F | GTACCAGATTACGCTCATATGATGAGAGCTCCAGATGAGGTGC |
| pGADT7-*Ss*-NOBP-1-R | CAGCTCGAGCTCGATGGATCCTTATCTCTTCTTACCACCACGTTTTC |
| pGADT7-*Ss*-PNO-1-F | GTACCAGATTACGCTCATATGATGGCAGATATGGATATACCAATGG |
| pGADT7-*Ss*-PNO-1-R | CAGCTCGAGCTCGATGGATCCTTATTTGTCTTGTATCGTATTATTAC |
| *Ss*-NOBP-1-HA-F | CCGTCAGATCCGCTAGCCACCATGAGAGCTCCAGATGAGGTG |
| *Ss*-NOBP-1-HA-R | ATCGTAAGGATATCTCGAGCCTCTCTTCTTACCACCACGTT |
| *Ss*-PNO-1-Myc-F | CCGTCAGATCCGCTAGCCACCATGGCAGATATGGATATACCAATGG |
| *Ss*-PNO-1-Myc-R | CAGCTTCTGCTCGCCGATCGCTCTGTCGAATGCTCTACTTGCATA |
| *Ss-nobp-1*-Prom-F | CAGGTCGACTCTAGAGGATCGAAGATATGCTTCAACAAC |
| *Ss-nobp-1*-Prom-R | GTTCTTCTCCTTTACTCATTGGCACCTCATCTGGAGCTCTCAT |
